# 

**Published:** 1860-01

**Authors:** 


### INDEX TO VOL. VII.

OF

### HALL'S JOURNAL OF HEALTH.

PAGE

Averting Disease. . . . . . . . . . . . . . . . . . . 80
Ash-Sifter . . . . . . . . . . . . . . . . . . . . . . 88
Athletics . . . . . . . . . . . . . . . . . . . . . . 145
Apples . . . . . . . . . . . . . . . . . . . . . . . . 233
Alexander, J. W., D.D. . . . . . . . . . . . . 241
Aim of Life . . . . . . . . . . . . . . . . . . . . 256
Action, A Kind . . . . . . . . . . . . . . . . . . 273
Benefits of Reading Aloud . . . . . . . . . 32
Bites, Poisonous . . . . . . . . . . . . . . . . 36
Burying Alive . . . . . . . . . . . . . . . . . . 38
Bread and Milk . . . . . . . . . . . . . . . . . 73
Bowels Regulated . . . . . . . . . . . . . 92, 271
Bathing . . . . . . . . . . . . . . . . . . . . . . 101
Breakfast Early . . . . . . . . . . . . . . . . . 116
Balance of Population . . . . . . . . . . . . 120
Best Doctors . . . . . . . . . . . . . . . . . . . 162
Breakfast Early . . . . . . . . . . . . . . . . . 216
Boys, Our . . . . . . . . . . . . . . . . . . . . . 217
Baby-Talk . . . . . . . . . . . . . . . . . . . . . 265
Causes of Disease . . . . . . . . . . . . . . . 38
Corns . . . . . . . . . . . . . . . . . . . . . . . . 67
Crazy People . . . . . . . . . . . . . . . . . . . 83
Costiveness . . . . . . . . . . . . . . . . . . . . 92
Clerical Marriages . . . . . . . . . . . . . . . 129
Central Park . . . . . . . . . . . . . . . . . . . 147
Cellars . . . . . . . . . . . . . . . . . . . . . . . 178
Catarrh . . . . . . . . . . . . . . . . . . . . . . . 189
Colds . . . . . . . . . . . . . . . . 37, 85, 189, 219
Children . . . . . . . . . . . . . 11, 184, 228, 231
Checking Perspiration . . . . . . . . . . . . 252
Consumption . . . . . . . . 45, 69, 72, 169, 261
Disease, Causes of . . . . . . . . . . . . . . . 38
Death-Bed Repentance . . . . . . . . . . . . 59
Depopulation . . . . . . . . . . . . . . . . . . . 106
Dying Nations . . . . . . . . . . . . . . . . . . 108
Donation Parties . . . . . . . . . . . . . . . . 121
Dyspepsia . . . . . . . . . . . . . . . . . . . 42, 160
Doctors, The Best . . . . . . . . . . . . . . . 162
Drinking Water . . . . . . . . . . . . . . . . . 184
Drunken Women . . . . . . . . . . . . . . . . 185
Domestics . . . . . . . . . . . . . . . . . . . . . 193
Drunkenness . . . . . . . . . . . . . . . 1, 42, 280
Eyes . . . . . . . . . . . . . . . . . . . . . 10, 39, 69

PAGE

Early Breakfast . . . . . . . . . . . . . . . . . 116
Each the Best . . . . . . . . . . . . . . . . . . 181
Eight to Sixteen . . . . . . . . . . . . . . . . 183
Eating . . . . . . . . . . . . . . . . . . . . . 94, 232
Electric Magnetism . . . . . . . . . . . . . . 234
Fruits, Use of . . . . . . . . . . . . . . . . . . 36
Flannel, Wearing . . . . . . . . . . . . . . . 72
Feet . . . . . . . . . . . . . . . . . . . . . . . . . 91
Fire on the Hearth . . . . . . . . . . . . . . 226
Farming . . . . . . . . . . . . . . . . . . . . . . 269
Green Persons . . . . . . . . . . . . . . . . . 18
Growing Beautiful . . . . . . . . . . . . . . 68
Gymnasiums . . . . . . . . 253, 152, 134, 109
Growing Old Happily . . . . . . . . . . . . 117
Garters, Wearing . . . . . . . . . . . . . . . 234
Hotel Life . . . . . . . . . . . . . . . . . . . . . 16
Hominy . . . . . . . . . . . . . . . . . . . . . . 20
Health Tracts . . . . . . . . . . . . . . . . . . 22
Health without Medicine . . . . . . . . . . 21
Husbands . . . . . . . . . . . . . . . . . . . . . 29
How to Walk . . . . . . . . . . . . . . . . . . 46
Hair-Wash . . . . . . . . . . . . . . . . . . . . 71
Hammer on . . . . . . . . . . . . . . . . . . . 141
Household Slaveries . . . . . . . . . . . . . 193
House Warming . . . . . . . . . . . . . . . . 226
Households Contrasted . . . . . . . . . . . 247
Happy Marriages . . . . . . . . . . . . . . . 267
Inconsiderations . . . . . . . . . . . . . . . . 35
Infants and Air . . . . . . . . . . . . . . . . . 108
Ice-Water . . . . . . . . . . . . . . . . . . . . . 173
Infant Night Management . . . . . . . . . 278
Living on Excitement . . . . . . . . . . . . 15
Living Yet . . . . . . . . . . . . . . . . . . . . 190
Lager Beer . . . . . . . . . . . . . . . . . . . . 216
Life's Maxims . . . . . . . . . . . . . . . . . . 223
Lunatics Gladdened . . . . . . . . . . . . . 285
Music Healthful . . . . . . . . . . . . . . . . 41
Manly Carriage . . . . . . . . . . . . . . . . 47
Moral Excitement . . . . . . . . . . . . . . . 61
Measles and Consumption . . . . . . . . . 69
Mistaken Benevolence . . . . . . . . . . . . 87
Marriage . . . . . . . . . . . . . . . . . . 267, 129
Manners . . . . . . . . . . . . . . . . . . . . . . 170

PAGE
Metamorphoses....................187
Miasma..........................204
Manual-Labor Schools.............219
Milk....................73, 112, 230
Magnetism.......................234
Matches.........................284
New Shoes Made Easy............. 67
National Dietetics.............. 76
Neglecting Colds................ 85
Nicholas of Russia.............. 89
Night Air.......................213
Nervousness.....................222
Olden Time......................104
Old Age Beautiful...............117
Our Boys........................217
Over-Eating.....................232
Position in Sleep............... 47
Phrenological Chair............. 45
Physiology...................... 57
Poisonous Rooms.................107
Panacea.........................113
Prostituted Health..............128
Parks, Public...................149
Periodical Literature...........241
Physical Training........134, 152, 253
Printers and Printing.........273, 277
Piano, the Worcester............282
Riding in Cars..................  8
Reading Aloud................... 32
Rules for Winter................ 44
Regimen.........................174
Retiring from Business..........256
Restless Wanderers..............281
Sleeplessness................... 25
Skating......................... 34
Spinal Deformities.............. 45
Successful Men.................. 63

PAGE
Sabbath Physiology.............. 70
Sour Stomach.................... 93
Spring Diseases.................118
Singular Medicine...............119
Sleeping in Church..............120
Sleep.................25, 47, 95, 175
Spitting Blood..................179
Small Bed-Chambers..............180
Slavery North and South.........193
Snapping up.....................225
Sleeping with the Old...........275
Strela Matches..................284
Too Late........................ 15
Traveling Hints................. 30
Three Health Essentials......... 73
Thrift and Health............... 79
Throat and Lungs................114
Teeth...........................150
Table Manners...................170
Tomatoes........................172
Tobacco Users...................179
Unitary Households.............. 53
Ways to Drunkenness.............  1
Winter Rules.................... 44
Walking......................... 46
Winter Shoes.................... 67
Wise Charities.................. 88
Worth Remembering.............. 90
Whisky Doctors..................124
What Killed Him.................132
Washington Irving...............133
Water Filters...................159
Water, Ice......................173
Wearing Garters.................234
Young Old People................ 41
Young and Poor..................284

